# Kaempferol Suppresses Carbon Tetrachloride-Induced Liver Damage in Rats via the MAPKs/NF-κB and AMPK/Nrf2 Signaling Pathways

**DOI:** 10.3390/ijms24086900

**Published:** 2023-04-07

**Authors:** Changyong Lee, Sik Yoon, Jeon-Ok Moon

**Affiliations:** 1 College of Pharmacy, Pusan National University, Busan 46241, Republic of Korea; qhrrn79@pusan.ac.kr; 2 Department of Anatomy, College of Medicine, Pusan National University, Yangsan 50612, Republic of Korea; sikyoon@pusan.ac.kr

**Keywords:** kaempferol, antioxidative, anti-inflammatory, hepatoprotective, NF-κB, MAPK, Nrf2, AMPK

## Abstract

Oxidative stress plays a critical role in the development of liver disease, making antioxidants a promising therapeutic approach for the prevention and management of liver injuries. The aim of this study was to investigate the hepatoprotective effects of kaempferol, an antioxidant flavonoid found in various edible vegetables, and its underlying mechanism in male Sprague-Dawley rats with carbon tetrachloride (CCl_4_)-induced acute liver damage. Oral administration of kaempferol at doses of 5 and 10 mg/kg body weight resulted in the amelioration of CCl_4_-induced abnormalities in hepatic histology and serum parameters. Additionally, kaempferol decreased the levels of pro-inflammatory mediators, TNF-α and IL-1β, as well as COX-2 and iNOS. Furthermore, kaempferol suppressed nuclear factor-kappa B (NF-κB) p65 activation, as well as the phosphorylation of Akt and mitogen-activated protein kinase members (MAPKs), including extracellular signal-regulated kinase, c-Jun NH_2_-terminal kinase, and p38 in CCl_4_-intoxicated rats. In addition, kaempferol improved the imbalanced oxidative status, as evidenced by the reduction in reactive oxygen species levels and lipid peroxidation, along with increased glutathione content in the CCl_4_-treated rat liver. Administering kaempferol also enhanced the activation of nuclear factor-E2-related factor (Nrf2) and heme oxygenase-1 protein, as well as the phosphorylation of AMP-activated protein kinase (AMPK). Overall, these findings suggest that kaempferol exhibits antioxidative, anti-inflammatory, and hepatoprotective effects through inhibiting the MAPK/NF-κB signaling pathway and activating the AMPK/Nrf2 signaling pathway in CCl_4_-intoxicated rats.

## 1. Introduction

**Figure 1:**
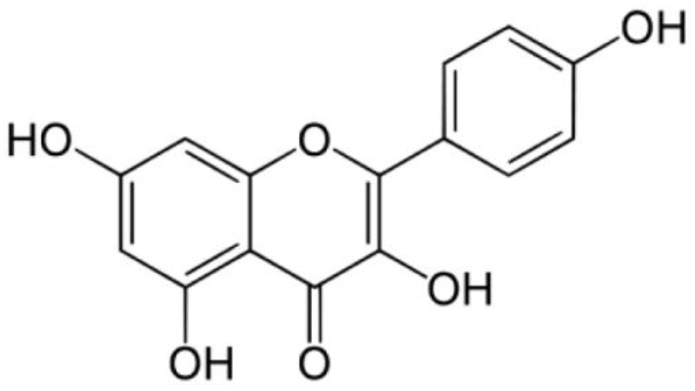
Structure of kaempferol.

Oxidative stress is implicated in the pathogenesis of various pathological conditions, including hepatic injuries [1,2,3]. The liver is susceptible to oxidative stress induced by several stimuli, such as alcohol, malnutrition, drugs, toxic substances, and hepatitis viruses. Oxidative stress causes hepatic injury by altering the structure or function of biomolecules and modulating molecular signaling pathways essential for regulating normal hepatic functions [4]. Hence, attenuating oxidative stress by administering antioxidants could be a rational therapeutic approach for the prevention or management of oxidative stress-related hepatic injuries [5]. Dietary phytochemicals have been shown to possess potent antioxidant properties [6,7]. Among the phytochemicals, flavonoids are considered the most active components present in various foods, vegetables, and herbal medicines. Kaempferol (3,5,7-trihydroxy-2-(4-hydroxyphenyl)-4H-1-benzopyran-4-one, Figure 1) is a flavonoid abundantly found in many edible and dietary sources, such as onion, pumpkin, cauliflower, carrot, broccoli, and black tea [8,9]. Approximately 17% of flavonoids in a regular diet are attributed to kaempferol [9]. In vitro, in vivo, and clinical studies have demonstrated that kaempferol and its glycosides exhibit a broad spectrum of pharmacological activities, including antioxidant, anti-inflammatory, anticancer, cardio-protective, and neuroprotective effects [10,11,12].

Numerous studies have reported that plant extracts containing kaempferol, such as *Miconia albicans*, *Onosma bracteata*, *Physalis peruviana*, and *Solanum elaeagnifolium*, exhibit protective effects in various experimental liver-injury models [13,14,15,16]. However, these extracts contain various phytochemicals in addition to kaempferol, such as quercetin, apigenin, and rutin, and those compounds have been shown to exhibit hepatoprotective effects. Therefore, it is essential to evaluate the hepatoprotective activity of kaempferol specifically, not of crude extracts. Recent studies have reported kaempferol’s hepatoprotective properties against: acetaminophen-induced liver damage by the upregulation/activation of silent information regulator 1 (SIRT1) signaling, alcoholic liver injury by attenuating the activity and expression of CYP2E1 and by enhancing the protective role of anti-oxidative defense system, and drug-induced hepatotoxicity in mice by inhibiting CYP2E1 activity [17,18,19]. Nevertheless, further research is necessary to elucidate the action mechanisms underlying kaempferol’s hepatoprotective properties as a modulator of signaling pathways related to oxidative stress-induced liver inflammation. Our experiments found that the oral administration of kaempferol has antioxidative, anti-inflammatory, and hepatoprotective effects on acute hepatic injury induced by carbon tetrachloride (CCl_4_) in rats. This paper aims to explore the molecular mechanisms of kaempferol in modulating oxidative stress and related signaling pathways. Specifically, we investigated the role of nuclear factor-kappa B (NF-κB), extracellular signal-regulated kinase (ERK), c-Jun NH_2_-terminal kinase (JNK), and p38 mitogen-activated protein kinases (MAPKs), as well as phosphatidylinositide 3-kinases (PI3K), protein kinase B (Akt), AMP-activated protein kinase (AMPK), and nuclear factor-E2-related factor (Nrf2), in the acute liver damage induced by CCl_4_ in rats.

## 2. Results

### 2.1. Antioxidative Activities of Kaempferol against FeSO_4_/H_2_O_2_-Induced Lipid Peroxidation and DPPH Radicals

Kaempferol was shown to possess potent antioxidant properties as demonstrated by its ability to inhibit FeSO_4_/H_2_O_2_-induced lipid peroxidation in rat liver homogenates. The well-known antioxidant, butylated hydroxytolune (BHT), was used as a positive control, and the IC_50_ of kaempferol was determined to be 9.69 μM, which was slightly higher than the IC_50_ of BHT at 8.66 μM (Table 1). The free-radical scavenging activity of kaempferol against the 2,2-diphenyl-1-picrylhydrazyl (DPPH) radical was also evaluated using Trolox as a positive control. Kaempferol exhibited high scavenging activity with an IC_50_ of 21.87 μM, while the IC_50_ of Trolox was 23.71 μM (Table 2).

### 2.2. Protective Effect of Kaempferol on t-Butyl Hydroperoxide (t-BHP)-Induced HepG2 Cell Damage

**Figure 2:**
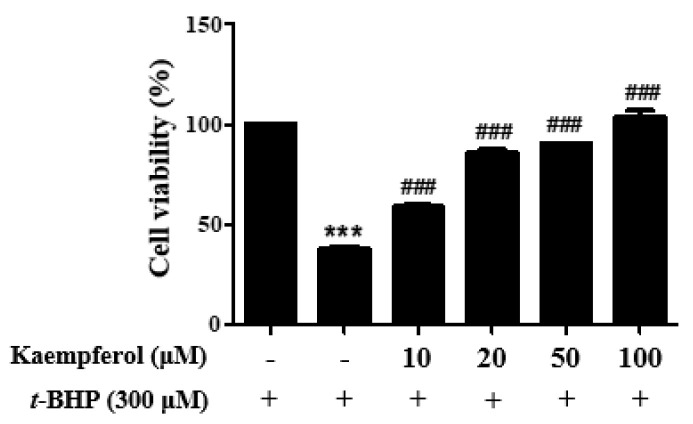
Effect of Kaempferol on the *t*-BHP-Induced HepG2 Cell Damage. HepG2 cells were pretreated with the indicated concentrations of kaempferol for 4 h and exposure to *t*-BHP (300 µM) for 3 h. Cell viability was estimated using the 3-(4,5-dimethylthiazol-2-yl)-2,5-diphenyltetrazolium bromide (MTT) assay. Values are mean ± standard error (*n* = 3). *** *p* < 0.001 vs. the control group and ^###^ *p* < 0.001 vs. the *t*-BHP treated cells.

*t*-BHP is a pro-oxidant agent that generates several reactive free radicals, which can cause cytotoxicity by disrupting normal cellular functions [20]. HepG2 cells are a well-established in vitro model for studying xenobiotic metabolism and liver toxicity, as they retain many specialized functions of normal human hepatocytes [21]. After exposure to 300 μM *t*-BHP for three hours, HepG2 cells showed a significant decrease in viability, with only 46% surviving compared to untreated cells. However, pretreatment with kaempferol provided a concentration-dependent protective effect against *t*-BHP-induced cell damage in HepG2 cells, with an EC_50_ of 45.8 μM (Figure 2). These results suggest that kaempferol has a hepatoprotective effect against *t*-BHP-induced cytotoxicity.

### 2.3. Changes in Body and Liver Weights and Serum Parameters in CCl_4_-Intoxicated Rats

**Figure 3:**
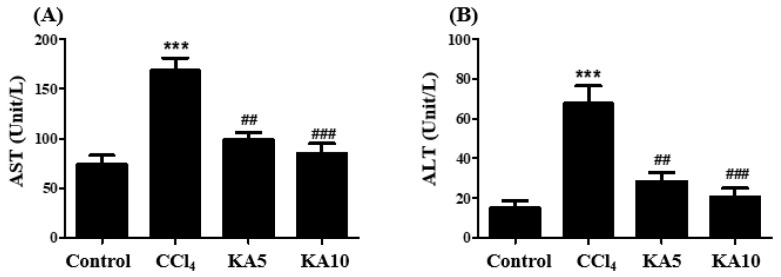
Effect of kaempferol on CCl_4_-induced serum parameter alterations. (**A**) Aspartate transaminase (AST); (**B**) Alanine transaminase (ALT). Groups are as described in “Materials and Methods”. Values are mean ± SE (*n* = 6). *** *p* < 0.001 vs. the control group, ^##^ *p* < 0.01, and ^###^ *p* < 0.001 vs. the CCl_4_ group.

The administration of CCl_4_ resulted in a significant increase in liver weight and liver/body weight ratio compared to the control group (Table 3). However, treatment with kaempferol effectively restored the enlarged liver induced by CCl_4_ to normal levels. To assess whether kaempferol protects the liver from CCl_4_-induced injury, we performed biochemical analyses of serum parameters (Figure 3). Our results showed that aspartate aminotransferase (AST) and alanine aminotransferase (ALT) activities were significantly increased in CCl_4_-intoxicated rats compared to the control group. However, kaempferol treatment dose-dependently reduced the levels of AST and ALT, indicating its in vivo hepatoprotective effects.

### 2.4. Liver Histopathology 

**Figure 4:**
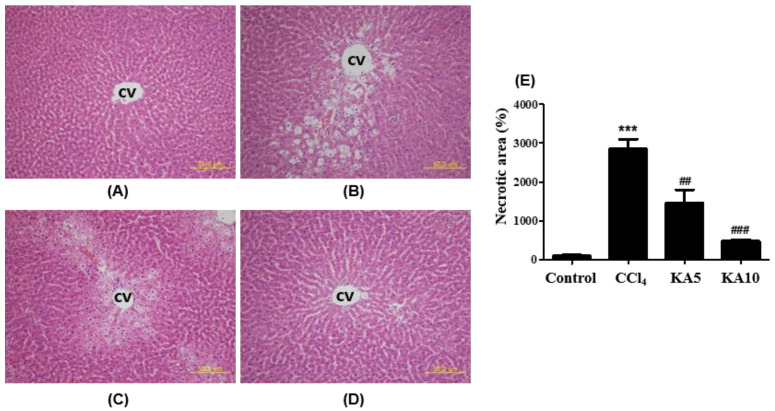
Effects of kaempferol on CCl_4_-induced histopathological changes in rat livers. H&E staining of liver sections from: (**A**) Control rats; (**B**) CCl_4_-treated rats; (**C**) CCl_4_-treated rats with kaempferol (5 mg/kg); (**D**) CCl_4_-treated rats with kaempferol (10 mg/kg). (**E**) Quantitative analysis of necrotic area observed on H&E-stained sections. *** *p* < 0.001 vs. the control group, ^##^ *p* < 0.01 and ^###^ *p* < 0.001 vs. the CCl_4_ group. CV: central vein. All images are original magnification ×400.

The effect of kaempferol on CCl_4_-induced histopathological alterations in the liver was evaluated by examining Hematoxylin and Eosin (H&E)-stained liver sections. The typical lobular architecture with central veins and radiating hepatic cords was disrupted, and there was sub-massive necrosis, vacuolization, and macrovesicular fatty changes in hepatocytes of CCl_4_-intoxicated rats (Figure 4B). However, the administration of kaempferol dose-dependently improved these pathologic changes and led to the restoration of normal cell integrity and hepatic architecture (Figure 4C,D). The quantitative analysis of the necrotic area observed on H&E-stained liver sections further supported kaempferol’s hepatoprotective activity against CCl_4_-induced liver damage.

### 2.5. Effects of Kaempferol on CCl_4_-Intoxicated Liver Inflammatory Mediators 

**Figure 5:**
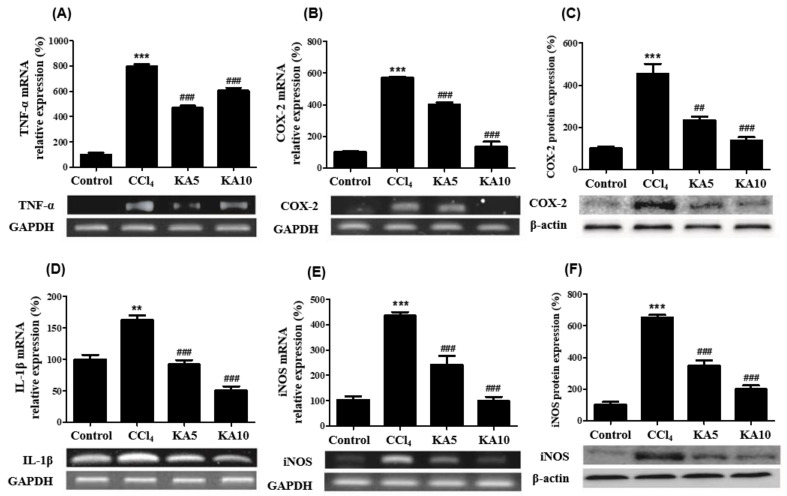
Effects of kaempferol on the pro-inflammatory mediators. The mRNA expression of TNF-α (**A**), IL-1β (**D**), COX-2 (**B**), and iNOS (**E**) in rat liver intoxicated with CCl_4_ was assessed using RT-PCR. Western blot analysis was performed to measure COX-2 (**C**) and iNOS (**F**) protein levels. Values are mean ± SE (*n* = 3). ** *p* < 0.01 and *** *p* < 0.001 vs. the control group, and ^##^ *p* < 0.01 and ^###^ *p* < 0.001 vs. the CCl_4_ group.

Reverse Transcription Polymerase Chain Reaction (RT-PCR) was utilized to measure the mRNA expression levels of the pro-inflammatory cytokine tumor necrosis factor (TNF)-α and Interleukin (IL)-1β in the liver. The results were quantified by normalizing against the housekeeping gene GAPDH’s mRNA expression. Kaempferol pretreatment significantly suppressed the CCl_4_-induced elevation of TNF-α and IL-1β expressions (Figure 5A,D). The mRNA expression levels of pro-inflammatory cyclooxygenase (COX)-2 and inducible nitric oxide synthase (iNOS), along with their protein levels in the liver, were measured through RT-PCR and Western blot analysis, respectively. Kaempferol administration significantly reduced the mRNA expression of COX-2 and iNOS (Figure 5B,E) as well as the corresponding protein levels in the CCl_4_-intoxicated rat liver (Figure 5C,F).

### 2.6. Effects of Kaempferol on MAPK/NF-κB Signaling Pathway in CCl_4_-Induced Liver Damages

**Figure 6:**
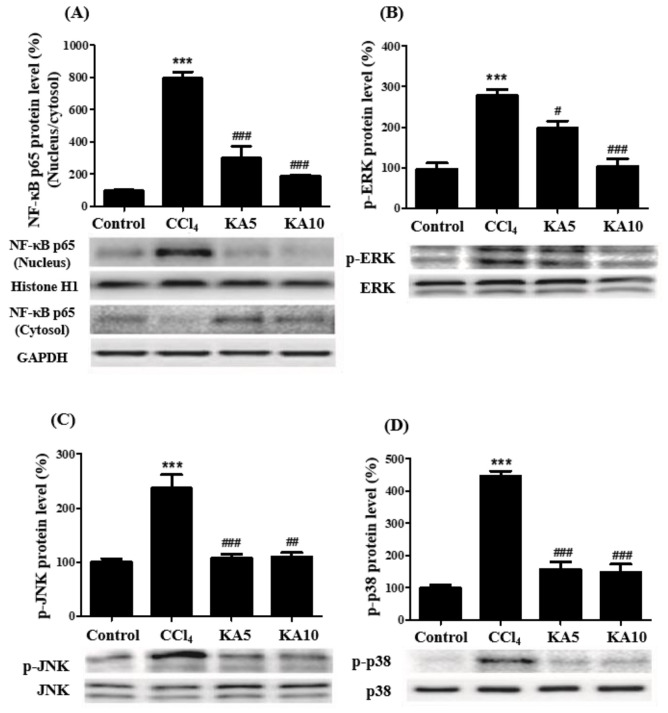
Effects of kaempferol on CCl_4_-induced NF-κB p65 activation (**A**) and ERK (**B**), JNK (**C**), and p-38 (**D**) phosphorylation. Western blotting was performed to detect the relative level of NF-κB p65 in the nuclear and cytosol and phosphorylated ERK, JNK, and p-38. Histone H1, GAPDH, and total ERK, JNK, and p-38 were used as loading controls, respectively. Values are mean ± SE (*n* = 3). *** *p* < 0.001 vs. the control group, and ^#^ *p* < 0.05, ^##^ *p* < 0.01, and ^###^ *p* < 0.001 vs. the CCl_4_ group.

Kaempferol’s effect on NF-κB activation was investigated by examining the translocation of NF-κB p65 from the cytosol to the cell nuclei. The protein levels of NF-κB in the nuclei and cytosol fractions were measured using Western blot analysis, with each fraction’s protein levels quantified by image analysis and normalized against histone H1 and GAPDH, respectively. As shown in Figure 6A, CCl_4_-intoxicated rat liver exhibited an increase in nuclear NF-κB p65 protein and a decrease in cytosolic NF-κB protein. However, kaempferol treatment significantly reduced the levels of translocated NF-κB in the nuclei fraction. 

To investigate the molecular mechanism of NF-κB activation in CCl_4_-intoxicated rats, the involvement of MAPK members was examined. MAPKs are activated by phosphorylation and transmit stimuli to a downstream target NF-κB. Western blot analysis was performed to measure the phosphorylated ERK1/2 (Figure 6B), JNK (Figure 6C), and p38 (Figure 6D) protein levels. The results showed that the phosphorylated protein levels of ERK1/2, JNK, and p38 MAPKs were elevated in the CCl_4_-treated rats. However, kaempferol treatment significantly decreased the phosphorylated protein levels of ERK1/2, JNK, and p38 MAPKs, indicating that kaempferol’s protective effect on CCl_4_-induced liver injury may be associated with the inhibition of NF-κB activation via the MAPK pathway.

### 2.7. Effect of Kaempferol on Oxidative Status in CCl_4_-Intoxicated Rat’s Liver

**Figure 7:**
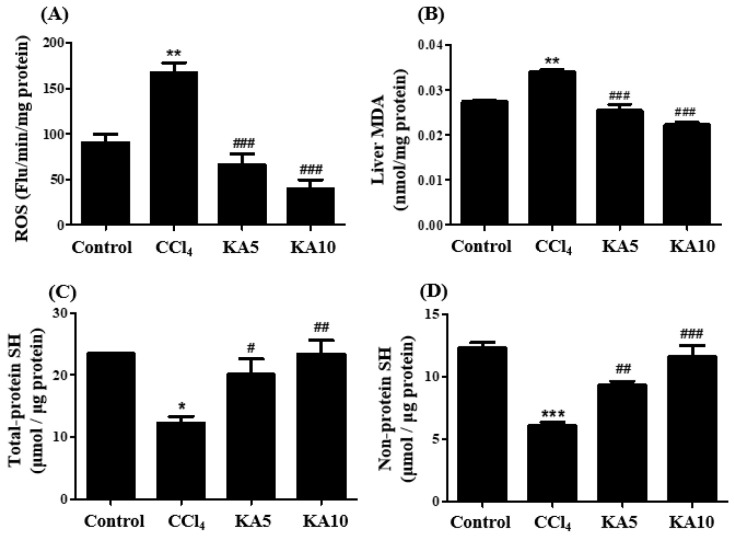
Effect of kaempferol on ROS (**A**), MDA (**B**), total SH (**C**), and non-protein SH (**D**) levels in rat livers intoxicated with CCl_4_. Values are mean ± SE of *n* = 6 rats/group. * *p* < 0.05, ** *p* < 0.01, and *** *p* < 0.001 vs. the control group, and ^#^ *p* < 0.05, ^##^ *p* < 0.01, and ^###^ *p* < 0.001 vs. the CCl_4_ group.

To assess the oxidative status, we measured the levels of reactive oxygen species (ROS), lipid peroxidation, and intracellular antioxidants in liver homogenates. The total ROS level was determined using a 2’,7’-dichlorofluorescein diacetate (DCFDA) probe, while the lipid peroxidation product MDA was measured to examine lipid peroxidation. As shown in Figure 7A,B, CCl_4_ treatment resulted in a significant increase in ROS levels and MDA amounts in the rat liver, indicating oxidative stress. However, kaempferol supplements dose-dependently suppressed these levels, highlighting its antioxidative properties. We also measured the levels of total SH (Figure 7C) and non-protein SH (glutathione, GSH) (Figure 7D), which are important endogenous antioxidants. In the CCl_4_-treated rat liver, these levels were reduced compared to the control group, but kaempferol prevented the reduction and restored the values almost to the extent of those of the control group, indicating its protective effects against oxidative damage.

### 2.8. Effects of Kaempferol on Nrf2 Activation and the PI3K/Akt and AMPK Signaling Pathways

**Figure 8:**
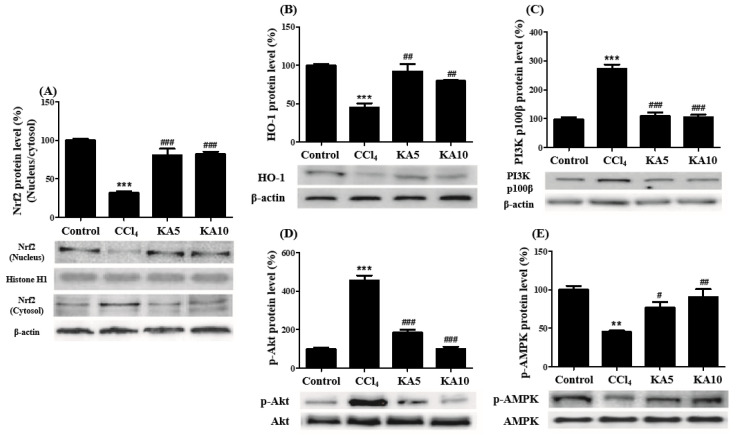
Effects of kaempferol on Nrf2 activation (**A**), HO-1 production (**B**), and PI3K level (**C**), and phosphorylated Akt (**D**) and AMPK (**E**) in CCl_4_-intoxicated rat liver. Western blotting was performed to detect these protein levels and quantified by image analysis. Histone H1, β-actin, total Akt, and AMPK were used as loading controls. Values are mean ± SE (*n* = 3). ** *p* < 0.01 and *** *p* < 0.001 vs. the control group and ^#^ *p* < 0.05, ^##^ *p* < 0.01, and ^###^ *p* < 0.001 vs. the CCl_4_ group.

We investigated the effect of kaempferol on Nrf2 activation by analyzing the protein levels of Nrf2 in the nuclear and cytoplasmic fractions of liver samples using Western blot analysis. We found that the ratio of Nrf2 protein in the nuclear-to-cytoplasmic fraction of CCl_4_-treated rat livers was significantly lower than that of the control group. Kaempferol treatment restored the ratio to near-control levels (Figure 8A). Additionally, we examined the protein levels of heme oxygenase (HO)-1, a downstream target gene of Nrf2. Our results show that kaempferol supplementation increased the protein levels of HO-1 in CCl_4_-intoxicated rat livers, confirming the activation of Nrf2 by kaempferol (Figure 8B). We further investigated the molecular mechanisms underlying kaempferol-induced Nrf2 activation by examining the protein levels of PI3K (Figure 8C), phosphorylated Akt (Figure 8D), and phosphorylated AMPK (Figure 8E). We found that the protein levels of PI3K and phosphorylated Akt were increased in CCl_4_-intoxicated rat livers, and kaempferol treatment suppressed these increases. In contrast, the reduced levels of phosphorylated AMPK in CCl_4_-intoxicated rat livers were restored by kaempferol supplementation.

## 3. Discussion

Oxidative stress occurs when there is an imbalance between the production of ROS and the body’s ability to detoxify and repair the damage caused by these reactive molecules. This stress is a key risk factor in the development of liver diseases. To investigate the mechanisms of hepatic injury and fibrosis, the hepatotoxicant CCl_4_ is commonly used. CYP2E1 converts CCl_4_ to a CCl_3_ radical, which reacts with molecular oxygen to generate CCl_3_OO• radicals. These highly reactive radicals can damage the hepatic endoplasmic reticulum’s phospholipids, initiating a chain reaction of lipid peroxidation [22]. This process results in membrane damage, a primary cause of CCl_4_-induced hepatocellular injury. 

Hepatocyte necrosis induced by CCl_4_ can be prevented by antioxidants that scavenge CCl_3_ and lipid peroxy radicals. Kaempferol, a natural flavonoid found in many plants, has been widely recognized for its potent antioxidant properties in numerous studies [8,9,10,11,12]. In our experiments, we observed that kaempferol exhibited antioxidant activity comparable to that of Trolox, a well-known antioxidant, in terms of its ability to scavenge DPPH radicals. Furthermore, kaempferol inhibited FeSO_4_/H_2_O_2_-induced lipid peroxidation to a similar extent as BHT, a known antioxidant. 

In this study, we evaluated the protective effects of kaempferol against liver injury induced by a single dose of CCl_4_ in rats. To assess the extent of liver injury, we measured the liver-to-body weight ratio and serum biochemical parameters, and we evaluated liver tissue morphology using H&E staining. Our results showed that CCl_4_ intoxication induced significant liver damage, as evidenced by changes in the aforementioned parameters. However, pretreatment with kaempferol at doses of 5 and 10 mg/kg in CCl_4_-intoxicated rats resulted in significant and dose-dependent improvements in these pathological alterations, suggesting a hepatoprotective effect of kaempferol against a CCl_4_-induced liver injury.

We evaluated the effect of kaempferol on CCl_4_-induced oxidative stress in the rat liver, as assessed by increased ROS production and decreased levels of total SH and non-protein SH. Our results show that kaempferol administration effectively attenuated CCl_4_-induced oxidative stress. Moreover, kaempferol was found to mitigate the high level of lipid peroxidation observed in CCl_4_-intoxicated rats, which indicates an imbalance between oxidative stress and antioxidant defense systems. These findings are consistent with the in vitro results, wherein kaempferol demonstrated significant hepatoprotective effects against *t*-BHP-induced oxidative stress on HepG2 cells, a commonly used cell line in liver research. Overall, our findings suggest that kaempferol possesses potent antioxidative activity, which is capable of protecting against oxidative stress and lipid peroxidation in both in vitro and in vivo systems.

This study aims to elucidate the molecular mechanisms underlying the hepatoprotective effects of kaempferol against CCl_4_-induced liver injury in rats. Specially, the study focuses on how kaempferol modulates the MAPKs/NF-κB and AMPK/Nrf2 signaling pathways, leading to anti-oxidative, anti-inflammatory, and hepatoprotective effects in CCl_4_-intoxicated rats.

Inflammation and elevated cytokines such as TNF-α and IL-1β are known to follow hepatocellular injury and are associated with the pathogenesis of liver diseases, in part, through the activation of the NF-κB signaling pathway. The production of pro-inflammatory mediators such as COX-2 and iNOS proteins, as well as TNF-α and IL-1β cytokines, is also regulated by this pathway [23]. The present study demonstrated that kaempferol supplementation effectively suppressed NF-κB activation in the liver and improved the profile of pro-inflammatory mediators, indicating that kaempferol exerts anti-inflammatory effects against CCl_4_-induced liver injury by inhibiting NF-κB activation.

The MAPK signaling pathway plays a crucial role in regulating diverse cellular processes, such as cell proliferation, differentiation, apoptosis, and stress responses [24]. Its three key members, ERK, JNK, and p38, can be activated by various stimuli, such as inflammatory cytokines and ROS, leading to the downstream activation of transcription factors, such as NF-κB, and the modulation of gene expression and cellular responses. In this study, we aimed to investigate the impact of kaempferol on the activation of MAPKs and the NF-κB pathway in a rat model of CCl_4_-induced liver injury. Our results demonstrate that CCl_4_ exposure led to the activation of ERK, JNK, and p38 MAPKs in the liver, but treatment with kaempferol attenuated this response. Notably, the kaempferol-mediated suppression of MAPK activity was associated with an inhibition of NF-κB signaling and reduced levels of pro-inflammatory mediators, such as TNF-α, IL-1β, COX-2, and iNOS. These findings suggest that kaempferol exerts its anti-inflammatory effects by interfering with the MAPK/NF-κB signaling axis. 

The Nrf2 signaling pathway plays a crucial role in protecting cells from oxidative stress by regulating the expression of various cytoprotective and detoxifying enzymes, which helps maintain cellular redox homeostasis [25]. One of the key functions of Nrf2 is the activation of the transcription of target genes that encode defense enzymes, such as HO-1 and GSH synthase/peroxidase, which can mitigate the damaging effects of oxidative stress. In this study, we investigated the effect of kaempferol on the Nrf2 pathway in CCl_4_-treated rats. Our results show that kaempferol treatment increased the translocation of Nrf2 into the nuclei and upregulated the expression of HO-1 in the liver. This suggests that kaempferol may enhance the cell defense system against oxidative stress by activating the Nrf2 pathway and promoting the expression of Nrf2 target genes, such as superoxide dismutase (SOD) and catalase, thereby reducing oxidative damage [26]. The improved oxidative status observed in CCl_4_-treated rats after kaempferol administration is likely due to a combination of Nrf2 activation and the direct antioxidative activity of kaempferol. Therefore, our findings suggest that kaempferol’s ability to activate the Nrf2 pathway and upregulate the expression of antioxidant enzymes may contribute to its overall antioxidant and hepatoprotective effects.

The regulation of Nrf2 by the PI3K/Akt pathway in response to cellular stress is well-established. Previous studies have shown that kaempferol activates the PI3K/Akt pathway to protect against oxidative stress in various models, such as Zearalenone-induced oxidative stress [27], isoproterenol-induced heart failure [28], and myocardial ischemia/reperfusion injury [29], by activating Nrf2. However, the specific effects of kaempferol on Akt can vary depending on cell type, tissue, and the type of damage. Other studies have demonstrated that kaempferol exerts its anti-inflammatory effects in cardiac fibroblasts [30] and reduces inflammation in an LPS-induced acute lung-injury model [31] by inhibiting Akt phosphorylation. It is worth noting that Akt activation has also been linked to NF-κB activation in other studies [32,33]. In our study, we found that CCl_4_ treatment increased the levels of PI3K and phosphorylated Akt in the liver, while kaempferol treatment reduced Akt phosphorylation. These findings suggest that Akt activation in this model is likely related to NF-κB activation, rather than Nrf2 activation. Our results indicate that the protective effects of kaempferol in the CCl_4_-induced liver injury model may be mediated through the suppression of Akt phosphorylation, leading to the inhibition of NF-κB activation. These findings provide further insights into the complex interplay between different signaling pathways involved in oxidative stress and inflammation.

The serine/threonine protein kinase AMPK has been widely reported as playing a critical role in regulating cellular stress and energy homeostasis [34]. Studies suggest that AMPK activation has a protective effect against oxidative stress and inflammation in various cell types, tissues, and organs. This protective potential is closely linked to the activation of Nrf2 signaling [35,36]. For instance, Velagapudi et al. demonstrated that kaempferol can activate the AMPK/Nrf2/HO-1 pathway in BV-2 microglia, inhibiting neuroinflammation [37]. Similarly, Du et al. found that kaempferol prevented Angiotensin II-induced cardiac fibrosis and dysfunction by modulating the AMPK/Nrf2 pathway [38]. In this study, we observed that kaempferol administration led to an upregulation of AMPK phosphorylation in CCl_4_-intoxicated rats, indicating the involvement of the AMPK/Nrf2 signaling pathway in the protection against oxidative stress and inflammation induced by CCl_4_.

## 4. Materials and Methods

### 4.1. DPPH Assay and Antioxidative Activity against FeSO_4_/H_2_O_2_-Induced Lipid Peroxidation

The radical scavenging activity of kaempferol was measured against stable DPPH free radicals using a published method [39]. To evaluate its effect on lipid peroxidation, rat liver homogenates (7.5 mg protein/mL) were treated with the Fenton reaction, which comprised 0.1 mM FeSO_4_ and 3 mM H_2_O_2_, along with various concentrations of kaempferol or BHT. The level of lipid peroxidation was determined as previous [40].

### 4.2. HepG2 Cell Damage Induced by t-BHP 

The HepG2 human hepatocellular carcinoma cell line was obtained from the Korea Cell Line Bank (Seoul, Republic of Korea) and was maintained in a DMEM medium supplemented with 10% fetal bovine serum, 1% glutamine, 0.01% penicillin, and 0.01% streptomycin at 37 °C with 5% CO_2_. Cells were seeded at a density of 2.0 × 10^4^ cells per well in 96-well plates and allowed to attach for 24 h. After serum starvation, cells were treated with various concentrations of kaempferol (10, 20, 50, and 100 μM) for 4 h, followed by exposure to 300 μM *t*-BHP for 3 h. Cell viability was assessed using the MTT assay, and the results were expressed as percentages of the viability of untreated cells.

### 4.3. Animals and Induction of Acute Liver Injury with CCl_4_

Male Sprague-Dawley rats weighing 140–160 g were obtained from Samtako (Osan, Republic of Korea). The animal protocol used in this study was reviewed and approved by the Pusan National University-Institutional Animal Care and Use Committee (Approval Number PNU 2016-1417) in accordance with ethical issues and scientific care. The rats were randomly divided into four groups (*n* = 6): Control, CCl_4_, KA5, and KA10. To induce liver injury, a single intraperitoneal (i.p.) injection of 25% (*w/v*) CCl_4_ (0.6 g/kg body weight) in olive oil was administered. Kaempferol (Sigma cat #K0133), suspended in a 0.5% sodium carboxymethylcellulose (CMC) solution was administered by oral gavage. The CCl_4_ group received CCl_4_ and CMC, while the KA5 and KA10 groups received CCl_4_ and kaempferol at 5 and 10 mg/kg body weight/day, respectively. The control group received olive oil and CMC. Kaempferol was treated twice, once at 16 h and once at 30 min before CCl_4_ intoxication. After 24 h of CCl_4_ injection, all rats were sacrificed under anesthesia, and blood samples were obtained from the inferior vena cava for biochemical analyses. The livers were excised and frozen in liquid nitrogen for further analysis.

### 4.4. Liver Histology and Biochemical Analysis of Serum Parameters 

Liver specimens were prepared for H&E staining following the methods described in our previous study [41]. The area of necrosis observed on the H&E-stained sections was quantitatively analyzed using Image J software (NIH, Bethesda, MD, USA). Serum levels of AST and ALT were measured using commercial kits (Asan Chemical Co., Cheonan, Republic of Korea) according to the manufacturer’s instructions.

### 4.5. MDA and ROS Level in Liver Tissues

To determine the levels of MDA in the liver, we followed the method described in our previous report [20,40]. The concentration of MDA was calculated based on the absorbance at 532 nm of the supernatant, using MDA tetrabutylammonium as a standard. We assessed the level of ROS by performing a fluorometric assay with DCFDA. The esterase and ROS (such as ∙O_2_^−^, ∙OH, and H_2_O_2_) in the sample oxidized DCFDA to the fluorescent 2’,7’-dichlorofluorescin, and we measured the change in fluorescence intensity with excitation and emission wavelengths set at 485 and 530 nm, respectively.

### 4.6. Total SH and Non-Protein SH Contents in Liver Tissues

To measure total SH, the liver tissue homogenate was mixed with 100 µL of 0.01 M 5,5-dithio-bis-2-nitrobenzoic acid, 4 mL methanol, and 1 mL 0.2 M Tris buffer (pH 8.2) and incubated at 25 °C for 15 min. After centrifugation at 1250× *g* for 30 min, the resulting supernatant was analyzed at 412 nm [42]. To determine non-protein SH, the homogenates were treated with trichloroacetic acid and centrifuged. One hundred μL of the resulting supernatant was mixed with 0.05 mL of 0.01 M NaNO_2_, 0.45 mL of 0.1 M H_2_SO_4_, and the mixture was allowed to stand for 5 min. Then, the sample was added to a solution containing 0.2 mL of 0.5% ammonium sulfamic acid, 0.1 mL of 1% mercuric chloride, and 0.9 mL of 3.4% sulfanilamide. Then, 1 mL of 0.1% N-naphthyl ethylenediamine in 0.4 M hydrochloric acid was added to the mixture. After 5 min, the mixture was measured at 540 nm using GSH as a standard.

### 4.7. Reverse Transcription-Polymerase Chain Reaction (RT-PCR) 

Total RNA was isolated from the tissue samples using Trizol reagent (Invitrogen, Carlsbad, CA, USA), and cDNA was synthesized from 1 μg of total RNA using the iScript cDNA Synthesis Kit (Bio-Rad, Hercules, CA, USA) following the manufacturer’s instructions. PCR was carried out using the Promega GoTaq Flexi DNA Polymerase PCR kit (Madison, WI, USA) with the following oligonucleotide sequences: TNF-α forward 5′-TTC TGT CTA CTG AAC TTG GGG GTG ATC GGT CC-3′, TNF-α reverse 5′-GTA TGA GAT AGC AAA TCG GCT GAC GGT GTG GG-3′, IL-1β forward 5′-ATG GCA ACT GTT CCT GAA CTC AAC T-3′, IL-1β reverse 5′-CAG GAC AGG TAT AGA TTC TTT CCT TT-3′, COX-2 forward 5′-CCA GAG CAG AGA GAT GAA ATA CCA-3′, COX-2 reverse 5′-GCA GGG CGG GAT ACA GTT C-3′, iNOS forward 5′-GAT TCA GTG GTC CAA CCT GCA-3′, iNOS reverse 5′-CGA CCT GAT GTT GCC ACT GTT-3′, GAPDH forward 5′-GAC AAC TTT GGC ATC GTG GA-3′, and GAPDH reverse 5′-ATG CAG GGA TGA TGT TCT GG-3′. The gene access numbers for TNF-α, IL-1β, COX-2, iNOS, and GAPDH were XM_03288689.1, NM_031512.2, NM_017232.3, L12562.1, and NM_017008.4, respectively. The mRNA levels were normalized using GAPDH as an internal control. The amplified products were separated on a 1.5% agarose gel and visualized using ethidium bromide staining under UV light illumination (Gel Doc/ChemiDoc Imager, Azure, Dublin, CA, USA).

### 4.8. Western Blot Analysis 

To prepare liver tissue samples for Western blotting, equal amounts of protein (30 μg) were extracted and resolved on 7–12% SDS-PAGE gels. The separated proteins were then transferred to PVDF membranes (Millipore) and incubated overnight at 4 °C with primary antibodies specific to the target proteins. The following day, the membranes were incubated with anti-mouse, anti-goat, or anti-rabbit secondary antibodies (Santa Cruz Biotechnology) for 1 h at room temperature. Primary antibodies used were COX-2 (sc-376861), iNOS (sc-7271), NF-κB p65 (sc-8008), p-ERK (sc-7383), p-p38 MAPK (sc-7973), p38 MAPK (sc-7972), p-Akt (sc-7985-r), Akt (sc-8312), PI3-kinase p110β (sc-602), Nrf2 (sc-722), HO-1 (sc-136961), AMPK (sc-25792), Histone H1 (sc-393358), and β-actin (Sc-47778), all purchased from Santa Cruz Biotechnology. Other primary antibodies used included ERK (#9102), p-JNK (#9255), JNK (#9252), and p-AMPK (#2535) from Cell Signaling Technology, and GAPDH (GTX100118) from Gene Tex. The blots were developed using an ECL detection kit (Advansta, CA, USA), and a quantitative analysis of protein levels was performed using ImageJ 1.53e software (NIH, Bethesda, MD, USA).

### 4.9. Statistical Analyses

Statistical analysis was performed using a one-way analysis of variance followed by Tukey’s multiple comparison test, and the results are presented as the mean ± standard error of the mean (SEM) from the indicated number of replicates. A *p*-value of less than or equal to 0.05 was considered statistically significant.

## 5. Conclusions

In conclusion, the results of this study demonstrate the significant potential of kaempferol as a protective agent against CCl_4_-induced acute liver damage. The observed inhibitory effects of kaempferol on NF-κB activation, pro-inflammatory cytokine expression (TNF-α and IL-1β), and protein production (COX-2 and iNOS) were associated with the suppression of upstream kinases such as ERK, JNK, p38 MAPKs, and Akt. Moreover, the administration of kaempferol effectively improved the oxidative balance in the livers of CCl_4_-intoxicated rats, likely through Nrf2 activation via AMPK phosphorylation. Overall, these findings suggest that kaempferol exerts antioxidative, anti-inflammatory, and hepatoprotective effects by modulating the MAPK/NF-κB and AMPK/Nrf2 signaling pathways in CCl_4_-intoxicated rats. These results support the potential of kaempferol as a therapeutic agent for mitigating liver inflammation induced by oxidative stress or hepatotoxins.

## Figures and Tables

**Table 1:** Antioxidative activities of kaempferol against the FeSO_4_/H_2_O_2_-induced lipid peroxidation.

Compound	Concentration (μM)	Inhibition (%)	IC_50_ (μM)
Kaempferol	2	0.34 ± 0.01	9.69
	5	17.94 ± 0.01	
	10	54.99 ± 0.00	
	20	75.80 ± 0.00	
BHT	2	15.39 ± 0.00	8.66
	5	27.33 ± 0.00	
	10	60.57 ± 0.02	
	20	70.05 ± 0.00	

Results are presented as mean ± standard error (SE) (*n* = 3). The inhibition (%) of malondialdehyde (MDA) formation by kaempferol or BHT was calculated based on the amount of MDA formation of the FeSO_4_/H_2_O_2_-treated control after subtracting the normal.

**Table 2:** Scavenging activities of kaempferol against the DPPH radical.

Compound	Concentration (μM)	Inhibition (%)	IC_50_ (μM)
Kaempferol	2	8.74 ± 0.01	21.87
	5	16.02 ± 0.39	
	10	23.29 ± 0.01	
	25	53.61 ± 0.00	
	50	73.31 ± 0.00	
BHT	5	15.00 ± 0.00	23.71
	10	20.56 ± 0.00	
	20	44.51 ± 0.00	
	50	83.36 ± 0.10	
	100	92.49 ± 0.07	

Results are expressed as mean ± SE (*n* = 3).

**Table 3:** Effects of kaempferol on the body and liver weights of rats treated with CCl_4_.

Compound	Bodyweight (g)	Liver Weight (g)	Ratio (%) ^a^
Control	154.02 ± 7.40	5.96 ± 1.25	3.85
CCl_4_	158.01 ± 7.29	7.69 ± 0.66 **	4.87
KA5	158.16 ± 4.62	6.98 ± 0.34 ^#^	4.42
KA10	156.75 ± 10.05	6.71 ± 0.29 ^#^	4.29

CCl_4_: CCl_4_-alone treated group; KA5: kaempferol (5 mg/kg) with CCl_4_; KA10: kaempferol (10 mg/kg) with CCl_4_. ^a^ Values are expressed as the ratios of liver/body weight. Data are the mean ± SE. (*n* = 6). ** *p* < 0.01 vs. the control group and ^#^ *p* < 0.05 vs. the CCl_4_ group.

## Data Availability

The data supporting the findings of this study are available within the article.
